# Coastal Ocean Metagenomes and Curated Metagenome-Assembled Genomes from Marsh Landing, Sapelo Island (Georgia, USA)

**DOI:** 10.1128/MRA.00934-19

**Published:** 2019-10-03

**Authors:** Julian Damashek, Christian F. Edwardson, Bradley B. Tolar, Scott M. Gifford, Mary Ann Moran, James T. Hollibaugh

**Affiliations:** a Department of Marine Sciences, University of Georgia, Athens, Georgia, USA; b Department of Marine Sciences, University of North Carolina at Chapel Hill, Chapel Hill, North Carolina, USA; University of Southern California

## Abstract

Microbes play a dominant role in the biogeochemistry of coastal waters, which receive organic matter from diverse sources. We present metagenomes and 45 metagenome-assembled genomes (MAGs) from Sapelo Island, Georgia, to further understand coastal microbial populations. Notably, four MAGs are archaea, with two *Thaumarchaeota* and two marine group II *Euryarchaeota.*

## Abstract (precis)

Microbes play a dominant role in the biogeochemistry of coastal waters, which receive organic matter from diverse sources. We present metagenomes and 45 metagenome-assembled genomes (MAGs) from Sapelo Island, Georgia, to further understand coastal microbial populations.

## ANNOUNCEMENT

Coastal oceans receive carbon and nutrients from rivers and marshes, driving high productivity. The metabolism of coastal microbes largely determines how much of the resulting organic matter (OM) is exported (1). Metagenomic data can provide insights into how microbial diversity relates to metabolic potential and drives OM processing (2). Coastal microbial biogeochemistry has been well studied at Sapelo Island, Georgia (3–5). Furthermore, these waters host a summer “bloom” of *Thaumarchaeota* and have been studied extensively to understand thaumarchaeal ecology (e.g., references 6–9). The metagenomic data presented here will guide an understanding of the microbial taxa in these waters and complement existing data for the same communities.

Seawater was collected at Marsh Landing (31°25′4.08″N, 81°17′34.26″W) as part of the Sapelo Island Microbial Carbon Observatory (http://www.simco.uga.edu/) by filtering through a 3.0-μm-pore-size prefilter and a 0.2-μm-pore-size Supor filter (Pall), which was frozen in liquid nitrogen (10). Duplicate filters were collected in August 2008 and 2009, 1 h before both day and night high tide on consecutive days (11). DNA extraction was done using the PowerSoil kit (Mo Bio), as described previously (7). DNA was sheared to ∼225 bp, and libraries were constructed with the TruSeq DNA kit (Illumina) at the Georgia Genomics and Bioinformatics Core. Replicates from day and night samples on consecutive days were pooled to make 4 libraries (08N, 08D, 09N, and 09D; see Table 1), which were sequenced on 25% of an Illumina HiSeq 2500 platform rapid lane (paired-end, 150-bp reads) at the HudsonAlpha Institute for Biotechnology.

**TABLE 1:** Sampling, pooling, and quality control of metagenomic libraries

Library	SRA BioSample no.	Pooled samples (filter IDs)^*a*^	Samples, collection date (mo/day/year), collection time^*b*^	No. of raw reads	No. of high-quality reads, with adapters removed	No. of trimmed reads	No. of paired reads
08D	SAMN12211998	FN64, FN65, FN74, FN75	FN64 and FN65, 8/6/08, 11:47; FN74 and FN75, 8/7/08, 11:03	5,569,551	5,393,758	4,496,340	3,590,532
08N	SAMN12212006	FN59, FN60, FN69, FN70	FN59 and FN60, 8/6/08, 00:15; FN69 and FN70, 8/7/08, 00:50	6,495,098	6,266,198	5,284,070	5,005,127
09D	SAMN12212021	FN143, FN144, FN153, FN154	FN143 and FN144, 8/12/09, 11:26; FN153 and FN154, 8/13/09, 13:59	6,258,053	6,028,226	5,049,333	4,240,004
09N	SAMN12212029	FN148, FN149, FN159, FN160	FN148 and FN149, 8/13/09, 01:14; FN159 and FN160, 8/14/09, 02:30	6,324,184	6,090,086	5,084,547	4,444,614
Total				24,646,886	23,778,268	19,914,290	17,280,277

a IDs, identifiers.

b For sampling details, see Gifford et al. (11).

Default software parameters were used, unless otherwise stated. The reads had adapters removed with Trim Galore (https://github.com/FelixKrueger/TrimGalore), were trimmed with PRINSEQ v.0.20.4 (12), and were joined using PEAR v.0.9.10 (13), using parameters described previously (14) (Table 1). Paired and high-quality orphaned/singleton reads were coassembled using metaSPAdes (“--meta”) within SPAdes v.3.7.0 (15), producing 83,626 contigs of >1,000 bp (*N*_50_, 718 bp; *L*_50_, 152,728; calculated with QUAST v.4.2 [16]).

Reads were mapped and indexed using Bowtie2 v.2.2.9 (17) and SAMtools v.1.3.1 (18), and contigs of >2.5 kbp (*n =* 18,714) were binned using anvi’o v.3 (19), following published protocols (20) (http://merenlab.org/data/tara-oceans-mags/). An anvi’o contig database was built to calculate k-mer frequencies, determine genes using Prodigal v.2.6.3 (21), and identify single-copy genes (22, 23) using HMMER v.3.1b2 (24). Bins generated by CONCOCT v.1.0.0 (25) were refined using the anvi’o interactive interface (26). Completeness and redundancy were assessed using anvi’o and CheckM v.1.0.12 (27); bins with <10% redundancy and ≥50% completeness were rerefined to minimize redundancy. Their resulting completeness and redundancy were estimated using anvi’o, CheckM, and the Microbial Genome Atlas (MiGA) Web server (28) (last accessed 18 August 2018). The resulting bins with completion of ≥50% were considered metagenome-assembled genomes (MAGs; *n =* 45) and were taxonomically annotated with MiGA. MAGs annotated below the order (genus) level included *Thaumarchaeota* (Nitrosopumilus spp., *n =* 2), marine group II *Euryarchaeota* (*n =* 2), *Synechococcaceae* (strain WH 8109, Cyanobium sp., *n = 2*), *Rhodobacteraceae* (Phaeobacter spp., *n =* 5), *Pelagibacteraceae* (*n =* 2), *Flavobacteriia* (*n =* 3), *Acidimicrobiaceae* (Ilumatobacter spp., *n =* 2), and *Halieaceae* (*n =* 1) (see https://figshare.com/articles/SIMO_MAG_table_v2/9791465/1).

### Data availability.

The reads, coassembly, and MAGs were deposited under GenBank BioProject number PRJNA552566. The reads are under SRA accession numbers SRX6421373 to SRX6421376. The coassembly and MAGs are under whole-genome sequencing (WGS) project numbers VMBT00000000 to VMDM00000000.
